# Defining severity of adhesions during redo cardiac surgery using Preoperative Computed Tomography scans and impact on surgical approach

**DOI:** 10.1186/1749-8090-10-S1-A219

**Published:** 2015-12-16

**Authors:** Tze Hung Siah, Marios E Patronis, Francesca Th'ng, Vipin Zamvar

**Affiliations:** 1 Medical Student, College of Medicine and Veterinary Science, 49 Little France Crescent, University of Edinburgh, Edinburgh, EH16 4SB, UK; 2 Specialty Doctor, Department of Cardiothoracic Surgery, Royal Infirmary of Edinburgh, Edinburgh, EH16 4SA, UK; 3 Surgical Research Fellow, Department of Clinical Surgery, University of Edinburgh, Edinburgh, EH16 4SA, UK; 4 Consultant Cardiothoracic Surgeon, Department of Cardiothoracic Surgery, Royal Infirmary of Edinburgh, Edinburgh, EH16 4SA, UK

## Background/Introduction

Mortality and morbidity of redo cardiac surgery is higher due to the presence of adhesions between cardiac structures and the chest wall. Preoperative Computed Tomography (CT) scans can help mitigate the risk of injury during redo surgery. Some surgeons use findings from preoperative CT scans to modify their surgical approach but its use is not universal.

## Aims/Objectives

We sought to determine if CT scans and time elapsed from initial cardiac surgery can predict the severity of adhesions, and whether CT scan findings are associated with the use of preventative surgical strategies.

## Method

We studied 92 patients referred for redo cardiac surgery. CT scan findings, operation notes findings, use of preventative surgical strategies, mortality and date of initial cardiac surgery were recorded.

## Results

In the study, 58 patients had preoperative CT scans and 34 patients did not. Preoperative CT scans identified 36 patients with moderate adhesions and 22 patients with severe adhesions. 13 out of 36 patients (33%) with moderate adhesions on CT scans had moderate adhesions during surgery. 16 out of 22 patients (73%) with severe adhesions on CT scans had severe adhesions during surgery. No association was found between the degree of adhesions on preoperative CT scans and surgical findings (p = 0.486). Severe adhesions on preoperative CT scans are not associated with greater use of preventative surgical strategies (p = 0.134). No significant difference in mortality was found in patients who had preoperative CT scan and those who did not (p = 0.070). No association was found between the severity of adhesions and time elapsed from initial cardiac surgery (p = 0.695).

## Discussion/Conclusion

Preoperative CT scans and time elapsed from initial cardiac surgery are not useful in predicting the severity of adhesions in redo cardiac surgery. Severe adhesions on CT scans are not associated with greater use of preventative surgical strategies.

